# An ethnomedicinal study of the Seri people; a group of hunter-gatherers and fishers native to the Sonoran Desert

**DOI:** 10.1186/s13002-015-0045-z

**Published:** 2015-08-11

**Authors:** Nemer E. Narchi, Luis Ernesto Aguilar-Rosas, José Jesús Sánchez-Escalante, Dora Ofelia Waumann-Rojas

**Affiliations:** 1 Centro de Estudios en Geografía Humana, El Colegio de Michoacán, Cerro de Nahuatzen 85. La Piedad, Michoacán, 59370 México; 2 N-Gen (Next Generation Sonoran Desert Researchers (http://nextgensd.com/)), Sonora, México; 3 CMMEX Herbarium, Instituto de Investigaciones Oceanológicas, Universidad Autónoma de Baja California, Carretera Ensenada-Tijuana No. 3917, Fraccionamiento Playitas. Ensenada, Baja California, 22860 México; 4 UNISON Herbarium, Departamento de Investigaciones Científicas y Tecnológicas de la Universidad de Sonora, Blvd. Luis Encinas y Rosales S/N, Col. Centro, Hermosillo, Sonora, 83001 México; 5 Marine Invertebrate Collection, Facultad de Ciencias Marinas, Universidad Autónoma de Baja California, Carretera Ensenada-Tijuana No. 3917, Fraccionamiento Playitas. Ensenada, Baja California, 22860 México

**Keywords:** Seri, Sonoran desert, Marine ethnomedicine, Knowledge acquisition, Gender division, Hunter-gatherer, Fisher

## Abstract

**Background:**

Worldwide, coastal communities’ ethnomedicinal knowledge has been sporadically recorded and poorly understood. Based on the ethnomedicinal knowledge of the Seri people; a hunting-gathering and fishing society of Northwestern Mexico, this study assesses a) the biological richness of Seri ethnomedicinal knowledge, b) the fidelity level of Seri remedies, and c) the association between gender, age, years of formal schooling and Seri ethnomedicinal knowledge.

**Methods:**

To assess the degree of ethnomedicinal knowledge proficiency, we conducted 75 open-ended semi-structured interviews collecting information on ethnomedicinal knowledge of marine and terrestrial organisms and the socio-demographic profile of each collaborator. With the support of primary collaborators, we collected the materials to be used as stimuli along our interviews. A correlation analysis was used to determine the relationship between gender, literacy and age with the ethnomedicinal knowledge proficiency. A paired t-test was used to determine differences in the number of remedies known by gender among members of the Seri community.

**Results:**

A total of 28 medicinal specimens were presented as stimuli material. Marine remedies (12 species), were represented by 4 algae, 3 mollusks, 3 echinoderms, on reptile, and one annelid. Terrestrial plants (13 species) were distributed in 12 families. About 40 % of marine preparations used the organism in whole. In contrast, 29 % of of the remedies involving plants made use of leafy branches. Stimuli materials are used against 17 ailments mainly, being diarrhea, colds, menstrual problems, and swelling the ailments against most organisms (44 %) are used for. Marine organisms presented higher fidelity level values overall, suggesting that lower fidelity levels in terrestrial plants reflect a process of continuous and ongoing experimentation with easily accessible biological materials. Highest fidelity level values were recorded for *Atriplex barclayana* (93.87 %) *Batis maritima* (84.37 %), and *Turbo fluctuosus* (84.21 %). Age moderately correlates to ethnomedicinal knowledge proficiency (r = 0.41). Conversely, years of formal schooling show a negative correlation with ethnomedicinal knowledge proficiency (r = -0.49). Significant differences (p <0.05) were observed on ethnomedicinal knowledge proficiency when gender groups were compared under a paired t-test.

**Conclusions:**

This research contributes to describing the complex biodiversity present in the ethnomedicinal systems of coastal non-agricultural societies. In addition, our research improves our understanding of the role that gender plays in the intra-cultural distribution of ethnomedicinal knowledge among Seri. Our results broaden our understanding of human adaptations to coastal and xeric environments. This research can potentially benefit the development of proposals to improve coastal and marine resource management and conservation while strengthening ethnomedicinal knowledge systems in populations, such as the Seri, limited by precarious socio-economic conditions and inadequate health services.

## Resumen

**Trasfondo:**

A nivel mundial, el conocimiento etnomédico comunidades costeras se ha registrado de forma esporádica. Basándonos en el conocimiento etnomédico del pueblo Seri; una sociedad de pescadores y cazadores-recolectores del noroeste de México, este estudio evalúa a) la riqueza biológica del conocimiento etnomédico Seri, b) el nivel de fidelidad de los remedios Seri, y c) la asociación entre género, edad y años de educación formal con el grado de conocimiento etnomédico alcanzado por individuos pertenecientes al grupo étnico Seri.

**Métodos:**

Para evaluar la competencia individual respecto al conocimiento etnomédico, realizamos 75 entrevistas abiertas y semi-estructuradas, que recopilaron información sobre el conocimiento etnomédico de organismos marinos y terrestres, así como el perfil sociodemográfico de cada colaborador. Con el apoyo de colaboradores primarios, colectamos los organismos a ser utilizados como materiales de estímulos a lo largo de nuestras entrevistas. Un análisis de correlación fue utilizado para determinar la relación entre género, edad y años de educación formal. Se utilizó una prueba t pareadas para determinar las diferencias en el número de remedios conocidos por los hombres en contraste con aquellos conocidos por las mujeres dentro de la comunidad Seri.

**Resultados:**

Un total de 28 especímenes medicinales se presentaron como material de estímulo. Los remedios marinos (12 especies), estuvieron representados por 4 algas, 3 moluscos, 3 equinodermos, un reptil, y un anélido. Las plantas terrestres (13 especies) se distribuyeron en 12 familias. En alrededor del 40 % de las preparaciones marinas se utiliza el organismo completo. En contraste, el 29 % de los remedios preparados con plantas se limitan al uso de ramas frondosas. Los materiales de estímulo se utilizan contra 17 enfermedades, siendo la diarrea, resfriados y problemas menstruales, las enfermedades combatidas por la mayoría de los organismos (44 %). Los organismos marinos presentan, en general, mayores niveles de fidelidad, lo que sugiere que las plantas terrestres están sujetas a una mayor experimentación dado su fácil acceso y colecta. *Atriplex barclayana* (93.87 %) *Batis maritima* (84.37 %), y *Turbo fluctuosus* (84.21 %) son los organismos con más altos niveles de fidelidad. La correlación entre edad y desempeño etnomédico es moderada (r = 0.41). Los años de educación formal presentan una moderada correlación inversa con el desempeño etnomédico (r = -0.49) Se observaron diferencias significativas (p <0.05) en competencia respecto al conocimiento etnomédico alcanzada por los grupos de colaboradores según su género y de acuerdo a los resultados arrojados por una prueba t pareada.

**Conclusiones:**

Esta investigación contribuye a describir la compleja biodiversidad presente en los sistemas etnomédico de las sociedades no-agrícolas costeras. Nuestra investigación mejora la comprensión del rol que el género juega en la distribución intra-cultural del conocimiento etnomédico Seri. Esta investigación alberga el potencial de beneficiar al desarrollo de propuestas encaminadas a mejorar el manejo costero y la conservación de recursos a la par de poder fortalecer los sistemas de conocimiento etnomédicos de poblaciones con condiciones socioeconómicas limitadas y servicios de salud inadecuados, como es el caso presente en la etnia seri.

## Background

The occurrence of ethnomedicines has been documented in virtually the entire world [1–7]. However, published research on marine ethnomedicine, is tremendously scarce [8]. A brief consultation of the Human Relations Area Files, one of the largest existing ethnographic collections, revels a meager number of ethnographies (N = 22) containing any mention of aquatic ethnomedicines [9]. Considering that ethobiological inventories are strongly delimited by biodiversity [10] and that humans consistently interact with marine biota in the most biodiverse coastal areas around the world [11], it is surprising to learn that there is a relatively low number of publications of marine ethnomedicines in the scientific literature.

Marine chemists, preoccupied with finding, describing, and isolateing novel compounds have provided their own explanations:

“The ocean lacks a marine ethnomedicinal history” ([12]: 271)

“Natural products from plants are often the cheapest and most effective drugs available, particularly in the Third World, and they come to us as a legacy of folk medicine based on herbal remedies. Unfortunately, we have no such legacy for the marine environment.” ([13]: 30)

“…due to technical barriers there has been a lack of extensive marine folk medicine in the western world.” ([14]: 16)

Such views are unsupported and incomplete. Firstly, there is enough evidence to argue for sustained and systematic human exploitation of marine resources since the dawn of the species [15, 16]. Secondly, it has been stated elsewhere that the use of marine ethnomedicines was already common around the globe since antiquity [17, 18]. Lastly, there is a long history of human seafaring and diving capabilities for extractive and belligerent purposes. The historical record of breath-hold diving goes as far back as 4500 BCE [19] and we know that this kind of diving has been practiced in various parts of the world ever since [20, 21]. There is enough documentation to even suggest the military use of diving bells as far back as 332 CE [22]. Currently, a considerable number of people worldwide make their living on breath-hold diving with little or no equipment at all [23–26]. Lastly, the number of research groups and publications working in ethnozoology, and particularly in marine ethnomedicine has increased considerably in the last decade [27, 28].

Previous studies on marine ethnomedicine have been mainly oriented to recording inventories of useful organisms at specific locations [29–35] or to describing the many uses of one single species [36, 37]. However, despite that ~40 % of the human world population lives within 100 km of a seashore [38], there is no certainty on how much human beings depend on marine organisms to provide themselves with health remedies.

The aforementioned is worrisome since: a) the trends of privatization [39, 40], and the consequences of global climate change, such as, rise in sea level [41, 42], and ocean acidification [43, 44], are displacing the most politically and economically underprivileged coastal populations and dispossessing them of relatively inexpensive marine resources which have always played an important part of their food and health systems [45, 46].

Hence, the main objective of this paper is to elucidate the roles that gender, age, and years of formal schooling play in the acquisition of Seri ethnomedicinal knowledge. The modern Seris (Comcaac) are descendants of the southernmost nomadic hunter-gatherer and fishing society of North America, native to the Central Gulf Region of the Sonoran Desert in western Sonora, Mexico. Their livelihood is still characterised for a marked seafaring tradition and an extensive use of marine resources [47]. In spite of active anthropological research among the Seri since the late 1800s [48] and to the best of our knowledge, published research linking Seri ethnobiological knowledge and socio-demographic variables is inexistent ([49] Stephen Marlett personal communication). Secondary objectives for this paper include: 1) highlighting the existence and importance of marine ethnopharmacies and 2) drawing attention on the complexity of ethnobiological and ethnoecological knowledge of coastal communities.

## Methods

### Study area

**Fig. 1:**
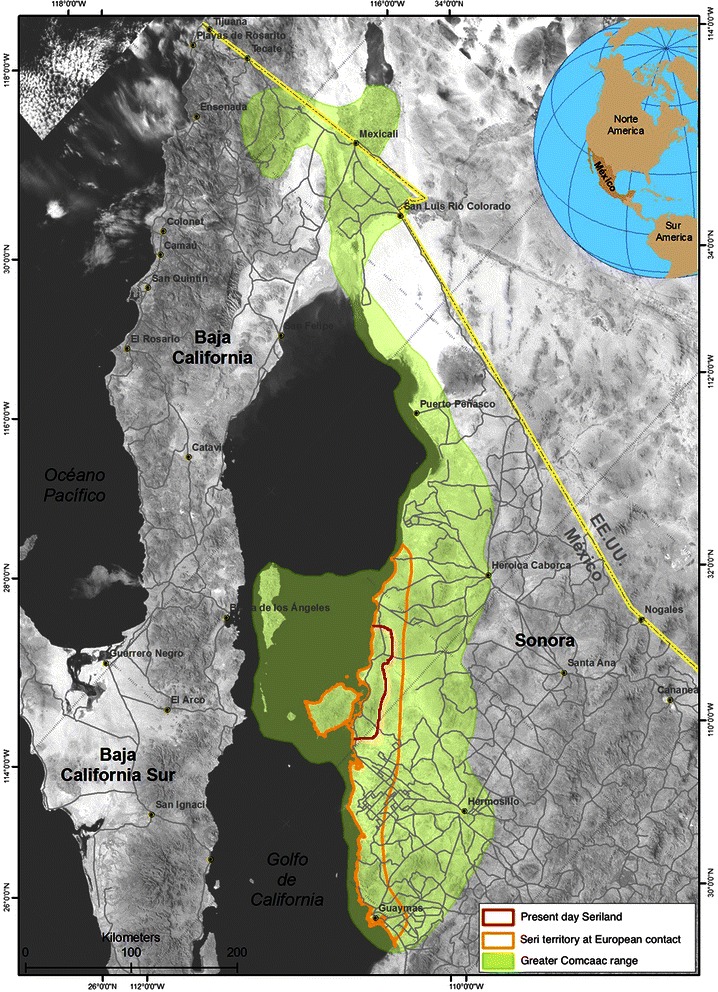
Seri territory. Location and boundaries of the present day Seri territory, the historical Seri territory at time of European contact, and the greater Comcaac range. Courtesy: Geovanni Cordero-Herrera

The study was carried out in present day Seri territory, in the central coastal portion of the state of Sonora, Mexico, between 26° 18′ and 32° 29′ N and 108° 25′ to 115° 03′ W. It starts north of Bahía Kino, and ends just north of Haxöl Iihom (Desemboque), and it includes the Tiburon and San Esteban Islands (Fig. 1).

There are two main Seri villages: Socaiix (Punta Chueca), located in the municipality of Hermosillo and Haxöl Iihom (Desemboque), in the municipality of Pitiquito.

The area is characterized by dry, rocky soils near the mountains and sandy soils in the valleys [50]. The annual pattern of temperature is high for the summer and below freezing nights-to-dawn during the winter. Highest daytime temperatures occur in summer and are usually below 40 ° C. In winter the predominant influence of northwesterly winds lowers the daily temperature to an average of 10 ° C [51].

The flora is a mixture of the typical Sonoran Plains type and a predominant Central Coast flora [52]. Felger and Moser [53] classified the dominant biotic communities in the area as marine vegetation (sea grass plains and algal communities), coastal scrub (mangrove and halophytic plain) desert scrub (coastal scrub, cactus, mesquite, riparian vegetation) and thorny shrubs (acacia).

Oceanographically, the Seri territory borders the Gulf of Californa, an interior basin of tectonic origin measuring 1130 km long and up to 209 km wide, presenting a maximum depth of around 3000 m [54, 55]. The Central Gulf Coast is characterized by the presence of an archipelago constituted by sills, channels, basins, and two notably large Islands: Ángel de la Guarda and Tiburon [56].

High biodiversity indices have been mentioned for virtually every taxa in the Sea of Cortez; i.e. plankton [57–59], nekton [60–62], benthos [63–65] and ornithos [66]. We will emphasize marine macro-fauna because previous studies [67, 68] and the present research suggest that this category represents the backbone of Seri marine medicine.

The total number of described macro-faunal species for the Sea of Cortez adds up to 5969 species. These estimates, excluding copepods and ostracods, account for 4854 species of invertebrates and 1115 species of vertebrates [63]. The most representative group of invertebrates are mollusks (2195 species), which account for 45 % of the known invertebrate species [56]. In terms of fisheries production, the yearly catch amounts for some 500 × 10^3^ t of seafood [69].

### Seri ethnobiological knowledge

The overall richness of the region has helped the Seri develop a large body of ethnobiological knowledge both in the marine and the terrestrial realms. Traditionally an indigenous group of hunter-gatherers and fishers, the Seri hold extensive knowledge of plants and animals living in their terrestrial [53, 67, 70–72] and marine [73–78] environments.

Seri ethnotaxonomy is a great example of the ample ethnobiological knowledge that these people have: the list of marine mollusk names collected by Cathy Moser Marlett [79] gathers some 290 different names for over 150 molluscan species; the work of Torre [73] and Torre and Findley [80] contains a hundred ichthyological ethnospecies comprehensive of fourteen *Chondrichthyes* and eighty-six *Osteichthyes.* Morales-Vera [74] has recorded one hundred and forty-five ethnoornitological species.

Seri use close to 90 different plant species for food purposes. These plants are found in a gradient that goes from completely terrestrial to supratidal, with some coastal shrubs and halophytes in between [53]. The use of plants is not limited to dietary necessities. Seri use marine and terrestrial plants for creating weapons [81], tools [78], adhesives [82], tanning materials [83], pigments [84], personal, ritual, and commercial adornment [85–88], musical instruments [89, 90], recreation supplies [91], storage items [92–94], shelter [95–97], fuel [98], and medicines [29, 53, 67, 99]. As in other settings [100] Seri ecological knowledge takes advantage of the many ecotopes that comprise their landscape. By using remedies from the desert, estuarine, and coastal habitats that surround them, Seris have built a robust and linguistically structured body of ethnoecological knowledge.^1^

There are no active specialists in Seri medical system [67]. In the past, only two specialists are known to have existed; Cola conáaij (midwife), who procured herbal and animal remedies from pregnancy to labor [101], and ziix haaco cama; a specialist skilled in spiritual support and supernatural technologies who administered no material remedy other than the ziix icóocmolca; a fetish rented to his patients as a protective charm [53]. Thus, the administration of Seri medicine is personal and individual, resulting in high variation and overlap in uses [53, 67]. Presently, the practice of traditional medicine is not as common as before and people rely heavily on the aid of medical doctors which they get by traveling outside of the community, to Puerto Libertad or Hermosillo, the state capital [53, 67, 99]. Nonetheless, a considerable number of women still engage in the preparation of traditional medicine, which they use to prepare balms, creams, and soaps to sell outside of the Seriland.

Women are also the major participants in picking up several fruits, wood, and raw materials for their baskets and handscrafts. On the contrary, it is men who spend most of the day fishing [53, 67, 102]. Some of their fishing expeditions require them to travel offshore and rely on whatever resources they can find on the Infiernillo Canal, the Midriff Islands or the Baja Californian shore [53].

Numerous works on Seri ethnobiological knowledge have already been carried out throughout the years. However, many of these interventions have relied on the participation of a limited number of indigenous collaborators. In the long run, it is only the opinions of these few Seri collaborators that have shaped what we think to know of Seri ethnobiological knowledge. Rentería-Valencia [personal communication] argues that the self-proclamation of these few collaborators as Seri knowledge experts has allowed them to amass a political power that still enables them to monopolize Seri research in ways that it is skewed to the outside observer who is not able to notice the slant *prima facie*.

After a long period of raids, submission attempts by the Spanish and Mexican Empires and a The Seris started integrating after 1920 when the incipient fisheries industry of Bahía Kino, Sonora demanded labor [102]. In 1926, given the U.S. prohibition of alcohol, the Kino Bay Sportsmen’s Club, was established with aims of developing recreational activities such as fishing, hunting, and exploring. The club members and their families provided the Seris with clothes, food, and money [53, 103]. The incoming gifts in addition to working in an industry that had always been part of the Seri livelihood convinced a number of Seris to settle nearby Bahía Kino. In 1938, the first fishing cooperative was formed in Bahía Kino with a majority of Seri fishers. The next year, Mexican fishers outnumbered the Seri inside the cooperative, causing tensions among the two groups and the founding member Jesús Solórzano, decided to move the cooperative 100 km north of Kino, at Haxöl Iihom [104]. Seris soon changed their seasonal patterns of subsistence migration and finally settled and opened up to surrounding neighbors and economies. The Mexican government saw his settling as a good opportunity to promote the acculturation policies started by anthropologist Manuel Gamio [105]. In order to do so, a number of people from different disciplines were deployed to Seriland to evaluate their health and infrastructural needs, as well as their degree of integration into the Mexican society [106, 107].

Presently, Seri economy is based on a mixture of commercial fisheries, sports-hunting, ecotourism, and wonderful handicrafts [87]. Fisheries, nonetheless, remain their greatest source of income and food [108–110]. However, and in spite of a relatively opened economy, the socio-economic status of the Seri people remains precarious and according to the National Population Bureau of Mexico, the degree of marginalization in their two settlements is high [111].^2^ Self-reported income for 2007-2008 averaged MEX$1600 a month, 4.4 times less than the national average income (MEX$7083) for that same period.

### Consent

The objectives of the project, along with the methods employed and the results expected were previously presented and discussed with the members of the community in two occasions. First, on October 14, 2008, when we described the project to the traditional authorities, Council of Elders, Seri Governor, and Presidente Ejidal, and primary collaborators. Second, on May 11, 2009, when we described the project and collaboration processes to secondary collaborators. Both meetings were held at Haxöl Iihom’s Guadalupe Victoria Elementary School. After these meetings each of the participants was given a written informed consent which was thoroughly explained individually. After reading the consent and making sure there were no more questions, each of the participants was asked to sign a facsimile copy of the written informed consent and keep the original for their own records.

### Data collection

The heterogenous nature of Seri ethnomedicinal knowledge made it necessary to ask for separate collection permits to three authorities. The National Commission for Aquaculture and Fisheries (Comisión Nacional de Acuacultura y Pesca - CONAPESCA) granted authorization for the sampling of marine organisms (authorization number DGOPA/08042/240709), The National Ministry for Natural Resources and the Environment (Secretaría del Medio Ambiente y Recursos Naturales - SEMARNAT) granted authorization for sampling insects (authorization number SGPA/DGVS/08424/08). Finally, the communal authority (Comisariado Ejidal) granted written permission for the researcher to use those plants previously collected by Seri healers as visual stimuli (letter dated May 29, 2009) under the conditions that: a) the plants were recognized as property of the Seri healers, b) the researcher could not transport the plants outside of the Seri territory, and c) the plants not consumed in preparing medicine would become part of a herbarium to be displayed at the Guadalupe Victoria elementary school of Haxöl Iihom.

The research was approved as ethical to human subjects by the University of Georgia Human Subjects Institutional Review Board on February 12, 2008, project number 2008-10528-0.

Data were collected in a yearlong field season starting August 2008 with the aid of seven primary collaborators. We considered primary collaborators as those people recognized as knowledgeable of Seri medicine by other people within their community. These collaborators helped to build a comprehensive database of organisms used medicinally by the Seri. To do so, each collaborator gave a free-list of marine and terrestrial organisms. The free-list served as a collection guide to gather the specimens, which we were able to collect with the aid of three of these collaborators; one man and two women, who provided more information on the medicines during our collecting trips. The rest of the primary collaborators were interviewed again later to carefully discuss the uses and modes of preparation for each of the remedies they had previously listed. The cultural relevance of the collected organisms was determined by using Smith’s saliency index processed with ANTHROPAC software [112]. The total number of organisms to be collected added up to a total of 50 terrestrial plants, 9 marine invertebrates, 7 marine algae, 4 halophytes, 2 fish, 1 marine reptile, and 1 seagrass.

### Algae and halophyte collection and identification

Specimens were found and collected in the intertidal zone on October 30, 2008 and April 3-13 2009, growing on sandy substrata and attached to rocks near Haxöl Iihom, Sonora, Mexico. Specimens were put in Ziploc™ bags and preserved in 4 % formalin seawater until further analysis in the laboratory at the Universidad Autónoma de Baja California (UABC), in Ensenada, Mexico. To identify the specimens, we compared our samples with the descriptions and illustrations by Dawson [113–115], Setchell and Gardner [116] and Yensen [117]. The collected specimens are housed at the UABC herbarium (CMMEX 10616 to 10632), which is included in the World Herbaria Index [118].

### Marine invertebrate collection and identification

Specimens were found and collected in the intertidal zone close to Haxöl Iihom, Sonora, Mexico on October 24, November 24, 2008, and April 3-13, 2009, in three different habitats; a) rocky shores, b) sandy beaches, c) mangrove estuary. Specimens were individually placed inside glass jars containing an 8 % formalin solution with enough volume to cover each organism until further analysis. Voucher specimens (CMEMM 5-6, 8, 11,13, 16, 18) are housed in the Laboratories of Invertebrate Zoology at UABC, in Ensenada, Mexico under the collection of marine ethnomedicines [29]. To identify the specimens, samples were compared to the descriptions and illustrations by Allen [119], Brusca [63], Fauchald [120], and Kudenov [121].

### Terrestrial plant collection and identification

The botanical specimens were collected on hillsides, arroyos, and desert plains surrounding Haxöl Iihom, Sonora, Mexico. Specimens were pressed and let to dry out with the aid of frequent wrap changes over a period of 3 weeks. Preserving botanicals in a xeric environment represents little risk of specimen destruction by fungi or molt. Plant specimens were mounted for permanent storage on sheets of ragbond paper along with their corresponding label. Since plants could not leave the community, species were identified in the field with the aid of primary collaborators and Felger & Moser’s notes on Seri ethnobotany [53, 67]. In addition and for corroborating the species classification, a high resolution photographic portfolio featuring each specimen was built along with their corresponding label containing information on a) the plant, b) a description of its appearance, c) a detailed description and geographical coordinates of the area where it was collected, and d) common, scientific, and emic name, was sent to one of the Authors (JJSE) for determination by comparison with the specimens stored at USON herbarium. As part of the research agreement with the community, the specimens are currently deposited at Escuela Primaria Guadalupe Victoria, Clave 26DPB0048X to let young Seri familiarize with their traditional pharmacy.

In order to build a visual stimuli portfolio short enough to allow for comfortable interviews we reduced the number of materials to be displayed, arbitrarily giving preference to marine organisms, since these were the main component of our broader research, and selecting 26 % of the total plant specimens randomly. The final *stimuli* portfolio included 4 algae, 4 halophytes, 7 marine invertebrates, 1 marine reptile, and 13 terrestrial plants.

A basal line of ethnomedicinal knowledge was developed with the aide of the primary collaborators. We asked each of the seven collaborators to narrate the mode of use and preparation of the selected stimuli material. The information was complemented with Felger and Moser’s notes [53]. Given that there was a radical variation among the descriptions of use and preparation given in 2008 by our collaborators and between these descriptions and those recorded by Felger and Moser back in 1974, Seri ethnomedicinal knowledge was assumed to be heterogenous and dynamic in the sense that Seri people are always experimenting with medicinal organisms to find new uses.

We prepared the collected organisms to be used as visual stimuli in carrying out a knowledge test among 68 consenting adults, 18 years old and above; 55 living in Haxöl Iihom and 13 residents of Socaaix.

To better observer the constant innovation within Seri ethnomedicinal knowledge, we structured the questionnaire as open-ended interviews. This decision also gave us an opportunity to capture all the diversity embedded in the permutations in Seri medicinal knowledge.

The questionnaire contained three components: 1) Seri name of the organism, and general characteristics of the environments where the organism is commonly found, 2) ethnomedicinal knowledge; what the organism is used for, and 3) sociodemographic data. The collaborators, while being presented with the visual stimuli, talked about each of the collected organisms by answering questions encompassing each of the components of the questionnaire in the form of slots and frames [122]; what is the name of X? What is X good for? We asked about self- assessed skills in using the plant: Have you personally prepared X? How do you prepare X for curing Y?

We created an identity profile for each collaborator by recording their age, gender, and years of formal schooling.

To compare the ethnomedicinal knowledge proficiency of each of the secondary collaborators in the realms of Seri ethnomedicine, we arbitrarily assigned each organism to one of two categories; marine or terrestrial depending on the ecosystem to which these organisms belong to.

### Data analysis

Data on collaborators’ backgrounds and Seri ethnomedicinal knowledge were entered in an electronic spreadsheet and organized for statistical analysis. The influence of age (18-79 years), and years of formal schooling (0-12 years) on ethnomedicinal knowledge proficiency was inferred with a correlation analysis at 95 % confidence level. Homogeneity in variance allowed for using an independent group t-test at 95 % confidence level between means to compare the differences on ethnomedicinal knowledge proficiency between men and women within the sample population.

### Calculating Seri ethnomedicinal knowledge

We arbitrarily assigned a value of 50 points to each of the first two components of the questionnaire in a way that if one collaborator knew the Seri names and modes of use of all of the organisms presented as visual stimuli, that person would get a 100 % score. However, the amount of variation, overlap and innovation within Seri ethnomedicine suggests there is no significant cultural consensus for Seri ethnomedicine. Therefore, for the final calculations on Seri ethnomedicinal knowledge, each of the answers on modes of use was adjusted by weighting (Wi) each answer with Friedman’s fidelity level [96] in the same way means are pondered in basic statistics. Friedman’s fidelity level was calculated with CONSENSUS2 a Matlab™ routine developed by one of the authors (NEN):

$$ \mathrm{F}\mathrm{l} = \mathrm{I}\mathrm{p}/\mathrm{I}\mathrm{u} $$

Where: Ip is the number of collaborators who in- dependently cited the importance of a species for treating a particular disease and Iu the total number of collaborators who reported the organism for any given disease.

The resulting formulas for calculating proficiency level in the ethnomedicinal knowledge test were:

$$ \mathrm{U}\mathrm{K} = {\displaystyle {\sum}^{\mathrm{n}}\mathrm{i}=1}{\mathrm{X}}_1{\mathrm{FL}}_{\mathrm{i}}/{\displaystyle {\sum}^{\mathrm{n}}\mathrm{i}=1}{\mathrm{W}}_{\mathrm{i}}=\left({\mathrm{X}}_{\mathrm{i}}{\mathrm{FL}}_{\mathrm{i}}+{\mathrm{X}}_2{\mathrm{FL}}_2+\dots +{\mathrm{X}}_{\mathrm{n}}{\mathrm{FL}}_{\mathrm{n}}\right)/{\mathrm{FL}}_1+{\mathrm{FL}}_2+\dots +{\mathrm{FL}}_{\mathrm{n}} $$

Where UK = Proficiency in knowing how organism is used.

X = Score for answer n regarding the use of the organism.

Fl_n_ = Fidelity level for organism n

$$ \mathrm{E}\mathrm{b}\mathrm{K} = \mathrm{T}\mathrm{K} + \mathrm{U}\mathrm{K} $$

Where

EbK = Ethnobiological knowledge proficiency.

TK = Proficiency in correctly naming the organism.

Total proficiency was calculated by adding the resulting EbK for terrestrial and marine knowledge.

## Results

### Seri ethnomedicinal knowledge

A wide diversity of organisms comprises the knowledge involved in treating human ailments in Seri culture. Throughout this research we used a total of 28 organisms spread across 6 Phyla (Table 1) to reflect upon this diversity.

**Table 1:** Frequency (F), average rank (AR), and salience (S) of free-listed organisms

Kingdom	Phylum	Scientific name	Seri Name	F	AR	S
			Literal translation^a^			
Animalia						
	Annelida	*Eurithoe cf. complanata* (Pallas 1766)	Xepenozatx	3.8	3.5	0.018
			Sting of the sea			
	Chordata	*Chelonia mydas* (Linnaeus 1758)	Moosni	3.8	5.50	0.014
			Unanalyzable			
	Echonodermata	*Heliaster kubinjii* (Xantus 1860)	Pyooque	13.2	2.29	0.098
			Unanalyzable			
		*Echinometra vanbruti* (A. Agassiz 1863)	Xepenosiml	79.2	1.4	0.694
			Marine barrel cactus			
		*Ophiocoma aethiops* (Lütken 1859)	Hanol cahít	1.9	5	0.004
			What cuts its arms			
	Molusca	*Modiolus capax* (Conrad 1837)	Satoj	1.9	6.00	0.009
			Unanalizable			
		*Octopus hubbsorum* (*Berry 1853*)	Hapaj cosni	39.6	2.76	0.245
			Octopi steak			
		*Turbo fluctuosus* (W. Wood, 1828)	Cotopis	22.6	2.33	0.142
			Suction cup			
Chromista						
	Chlorophyta	*Codium simulans* (Setchell and Gardner 1924)	Tacj oomas	5.7	4.33	0.036
			Bottle nose dolphin’s fishing line			
	Ochrophyta	*Sargassum sinicola* (Setchell and Gardner 1924)	Xpanams caacöl	1.9	2.00	0.014
	Ochrophyta	*Colpomenia tuberculata* (Saunders 1898)	Xpeetc	1.9	9.00	0.002
			Unanalyzable			
	Rhodophyta	*Kallymenia pertusa* (Setchell and Gardner 1924)	Moosni ipnáail	5.7	2.33	0.041
			The turtle’s skirt			
Plantae						
	Family					
	Aizoaceae	*Sesuvium sp*. (Linnaeus 1759)	Spitj Caacöl	1.6	21	0.005
			Large spitj			
	Apocynaceae	*Vallesia glabra* (Cavanilles 1724)	Tanoopa	1.6	2	0.013
			Unanalyzable			
	Asteraceae	*Ambrosia salsola* ((Torr. & A. Gray) Strother & B.G. Baldwin 1849)	Caasol cacat	20.6	6.08	0.139
			Large caasol			
	Bataceae	*Batis maritima* (Linnaeus 1759)	Pajoocsim	1.9	7	0.006
			Unanalyzable			
	Burseraceae	*Bursera microphylla* (Gray 1861)	Xoop	50.8	5.13	0.313
			Unanalyzable			
		*Bursera hindsiana* (Engler 1883)	Xopinl	9.5	5.50	0.048
			Xoop’s hand			
	Celastraceae	*Maytenus phyllanthoides* (D. Dietrich 1844)	Cos	12.7	7.25	0.072
			Unanalyzable			
	Chenopodiaceae	*Suaeda sp.* (Forsskål ex J. F. Gmelin, 1776)	Hatajípol	3.2	18.5	0.007
			Unanalyzable			
		*Atriplex barclayana* (D. Dietrich 1852)	Spitj	3.2	12.50	0.016
			Unanalyzable			
	Euphorbiaceae	*Acalypha californica* (Benthham 1844)	Queejam iti hacniix	38.1	5.50	0.228
			Which piles out of season			
	Malpighiaceae	*Callaeum macropterum* (D.M. Johnson 1986)	Haxz ooxmoj	4.8	4	0.031
			Dog’s hip			
	Malvaceae	*Sphaeralcea ambigua var. ambigua* (A. Gray 1887)	Jcoa ctamöc	25.4	5.56	0.169
			Male Jcoa			
	Menispermaceae	*Cocculus diversifolius* (*A*. de Candolle 1817)	Comixaz	22.2	5.79	0.128
			Unanalyzable			
	Rhizophoraceae	*Rhizophora mangle* (Linnaeus 1753)	Xnazolcam	26.4	3.93	0.100
			Unanalyzable			
	Verbenaceae	*Lippia palmeri* (S. Watson 1889)	Xomcahiift	39.7	6.24	0.218
			Unanalyzable			
	Viscaceae	*Phoradendron californicum* (Nuttall 1847)	Eaxt	6.3	6.25	0.022
			Unanalyzable			
	Zygophythaceae	*Larrea divaricata subsp. tridentata* (*DC.) Felger & C.H. Lowe (1970)*	Haaxat	66.7	3.67	0.499
			Unanalyzable			

^a^All translations taken from Moser and Marlett’s Seri Dictionary [161]

Marine remedies, amounting to 12 species, were represented by algae (33.3 %), halophytes (33.3 %) mollusks (25 %), echinoderms (16.6 %), reptiles (8.3 %), and annelids (8.3 %) Plants specimens (13 species), were distributed in 12 families. The family Burseracea was represented by the highest number of species 2. The vast majority of these plants (59 %) were shrubs, vines and trees were used in the same proportion (18 %), and herbs (6 %) were the least numerous.

### Parts used for remedy preparation

**Fig. 2:**
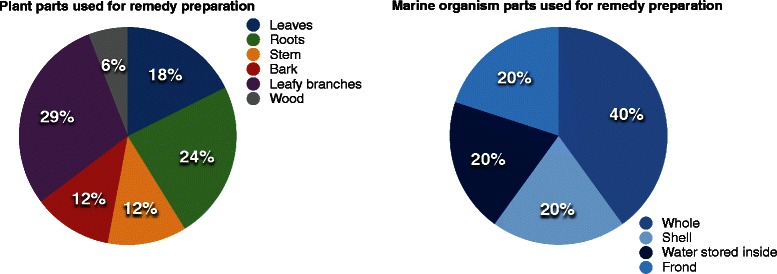
Parts used for remedy preparation

The organisms’ parts used for preparation are many (Fig. 2). In regards to marine organism (including algae), nearly 40 % of the preparations used whole organisms. The rest of the preparations involve the frond (20 %), shell (20 %), and even the seawater stored inside the organism (20 %). Plant parts used for preparation of remedies are far more heterogenous. The majority (29 %) make use of leafy branches, followed by roots (24 %) and leaves (18 %), stems and bark (12 % each), and finally, wood (6 %). Freshly harvested plants are preferred, but it is not uncommon for people to store dried bundles of *Larrea divaricata* or *Lippia palmeri.* Regarding marine organisms, most are used fresh, with the exception of echinoderms and shelled mollusks, which can be left to dry until needed.

### Medicinal uses of the organisms

The stimuli materials are used against 17 ailments, and one is used as sunblock. Diarrhea, colds, menstrual problems, and swelling are the ailments for which most of the organisms (44 %) are used against. The consensus on the main use of these organisms is greater for marine than terrestrial; more people use these organisms for the same specific purpose. Based on the fidelity level analysis (Table 2), there is, on average, more agreement on the uses given to marine organisms (mean Fl = 71.31) than the agreement found when mentioning plant uses (mean Fl = 36.82). Conversely, our collaborators preferred to mention any use for plant medicines (∑ = 645 uses) than to give their opinion on the use of marine medicines (∑ =533 uses). A relatively large number of organisms (41.37 %) were reported to have a same major use different from that previously reported in the literature [26, 48, 62, 63].

**Table 2:** Organisms, scientific name, Seri name, voucher number, most common use, and fidelity level

Kingdom	Phylum	Scientific name	Seri Name	Voucher number	Use	Ip	Iu	Fidelity level
Animalia								
	Annelida	*Eurithoe cf. complanata* (Pallas 1766)	Xepenozátx	CMEMM-13	Prepared in a tea in order to cease menstrual flow	2	3	66.66
	Chordata	*Chelonia mydas* (Linnaeus 1758)	Moosni	------	The oil of *C. mydas* is taken in a tablespoon as expectorant^a^	16	24	66.66
	Echonodermata	*Heliaster kubinjii* (Xantus 1860)	Pyooque	CMEMM-18	Prepared in a tea in order to cease menstrual flow and stop post-partum hemorrhage	8	10	80
		*Echinometra vanbruti* (A. Agassiz 1863)	Xepenosiml	CMEMM-8	Prepared into a tea to stop menstrual flow	37	46	80.43
		*Ophiocoma aethiops* (Lütken 1859)	Hanol cahít	CMEMM-16	Scorched and macerated into a paste applied to swollen areas	4	7	57.14
Mollusca								
		*Modiolus capax* (Conrad 1837)	Satoj	CMEMM-6	Shell is grounded, mixed with water and applied to the umbilicus of an infant to make it heal faster	2	6	33.33
		*Octopus hubbsorum* (*Berry 1853*)	Hapaj cosni	CMEMM-5	Crushed and cooked with dock (*Rumex*) and drunk to run faster	22	36	61.11
		*Turbo fluctuosus *(W. Wood 1828)	Cotopis	CMEMM-11	Shell is grounded, mixed with water and applied to the umbilicus of an infant to make it heal faster	16	19	84.21
Chromista								
	Chlorophtyta	*Codium simulans* (Setchell and Gardner 1924)	Tacj oomas	CMMEX 10616	Eyewash applied simply by soaking the algae in seawater and squeezing directly into the eyes^a^	13	26	28.57
	Ochrophyta	*Sargassum sinicola* (Setchell and Gardner 1924)	Xpanams caacöl	CMMEX 10620	The frond is boiled in freshwater, resulting in a tea that prevents epilepsy^a^	2	13	6.66
		*Colpomenia tuberculata* (Saunders 1898)	Xpeetc	CMMEX 10626	Water retained inside bulbose algae is drunk to cure dehydration, headache and light headedness^a^	2	10	20
		*Kallymenia pertusa* (Setchell and Gardner 1924)	Moosni ipnáail	CMMEX 10622	Heatened beneath a stone near a campfire, the frond is used as a cataplasm in swollen areas of the body^a^	3	6	50
Plantae								
	Family							
	Aizoaceae	*Sesuvium sp*. (Linnaeus 1759)	Spitj Caacöl		*Sesuvium* is used to wash stingray wounds^a^	5	12	41.66
	Apocynaceae	*Vallesia glabra* (Cavanilles 1724)	Tanóopa	USON 20152^b^	Leaves are toasted and macerated, the resulting powder is applied to control the itch of a rash, measles or chickenpox	3	9	33.33
	Asteraceae	*Ambrosia salsola* ((Torr. & A. Gray) Strother & B.G. Baldwin 1849)	Caasol cacat	No specimen	A tea made with the stem is used to heal swollen parts of the body^a^	12	39	30.76
	Bataceae	*Batis maritima* (Linnaeus 1759)	Pajóocsim		Macerated in water will cure diarrhea^a^	27	32	84.37
	Burseracea	*Bursera microphylla* (Gray 1861)	Xoop	USON 03688, 20051, 04998	Sap is used as sunblock^a^	12	53	22.64
		*Bursera hindsiana* (Engler 1883)	Xopinl	USON 03708	In a mixture with *Hyptis emoryi* it is prepared in a tea to treat colds.	19	38	50
	Celastraceae	*Maytenus phyllanthoides* (D. Dietrich 1844)	Cos	CMMEX 10631	A tea prepared with the leaves is used for a sore throat	17	40	42.5
	Chenopodiaceae	*Suaeda sp.* (Forsskål ex J. F. Gmelin, 1776)	Hatajípol	CMMEX 10630	A tea made from the roots is used to treat colds	19	30	63.33
		*Atriplex barclayana* (D. Dietrich 1852)	Spitj	CMMEX 10632	It is used in concoction in conjunction with *B. microphylla* against the painful sting of rays and skates	46	49	93.87
	Euphorbiaceae	*Acalypha californica* (Benthham 1844)	Queejam iti hacniix	USON 03709, 05377	The plant is let in water for a night. The water is then used to wash the head in presence of neuralgia	10	16	62.5
	Malpighiaceae	*Callaeum macropterum* (D.M. Johnson 1986)	Haxz ooxmoj	USON 09454	The tea extracted from this plant is most commonly used against diarrhea	9	45	60
	Malvaceae	*Sphaeralcea ambigua* Gray *var. ambigua* (A. Gray 1887)	Jcoa ctamöc	No specimen	The inner bark and pulp are poinded and made into a tea to cure sores in the mouth.	12	18	66.66
	Menispermaceae	*Cocculus diversifolius* (*A*. de Candolle 1817)	Comíxaz	ARIZ 356537	Used to prepare an infusion to treat diarrhea^a^	4	11	36.36
	Rhizophoraceae	*Rhizophora mangle* (Linnaeus 1753)	Xnazolcam	CMMEX 10627	A concoction of the plant is used against diabetes^a^	20	44	45.45
	Verbenaceae	*Lippia palmeri S.* (S. Watson 1889)	Xomcahíift	USON 02168, 04159	A tea prepared with the leaves is good against colds^a^	29	60	48.33
	Viscaceae	*Phoradendron californicum* (Nuttall 1847)	Eaxt	No specimen	A tea prepared with the leaves is used against diarrhea^a^	19	30	63.33
	Zygophythaceae	*Larrea divaricata subsp. tridentata* ((DC.) Felger & C.H. Lowe 1970)	Haaxat	USON 20038	The concoction of this plant is commonly used to treat smelly feet^a^	14	62	22.58

^a^uses different to those previously reported

^b^Voucher specimens to which collections were compared

### The distribution of Seri ethnomedicinal knowledge

**Fig. 3:**
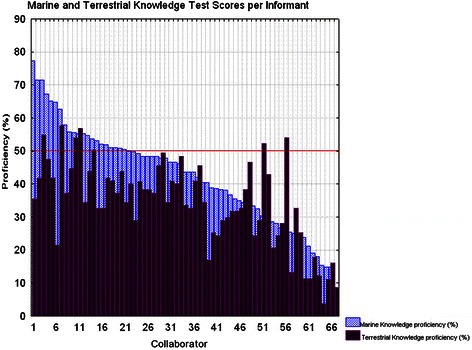
Ethnomedicinal knowledge test score per informant

At first glance, the results seem to show no difference between marine and terrestrial ethnomedicinal knowledge (Fig. 3). In most of the cases, the proficiency of each collaborator shows some degree of correspondence between what they know of marine medicine and what they know of terrestrial medicine. Whenever collaborators displayed a higher degree of knowledge in one of the two medicinal realms, they display some level of mastery in the other.

**Fig. 4:**
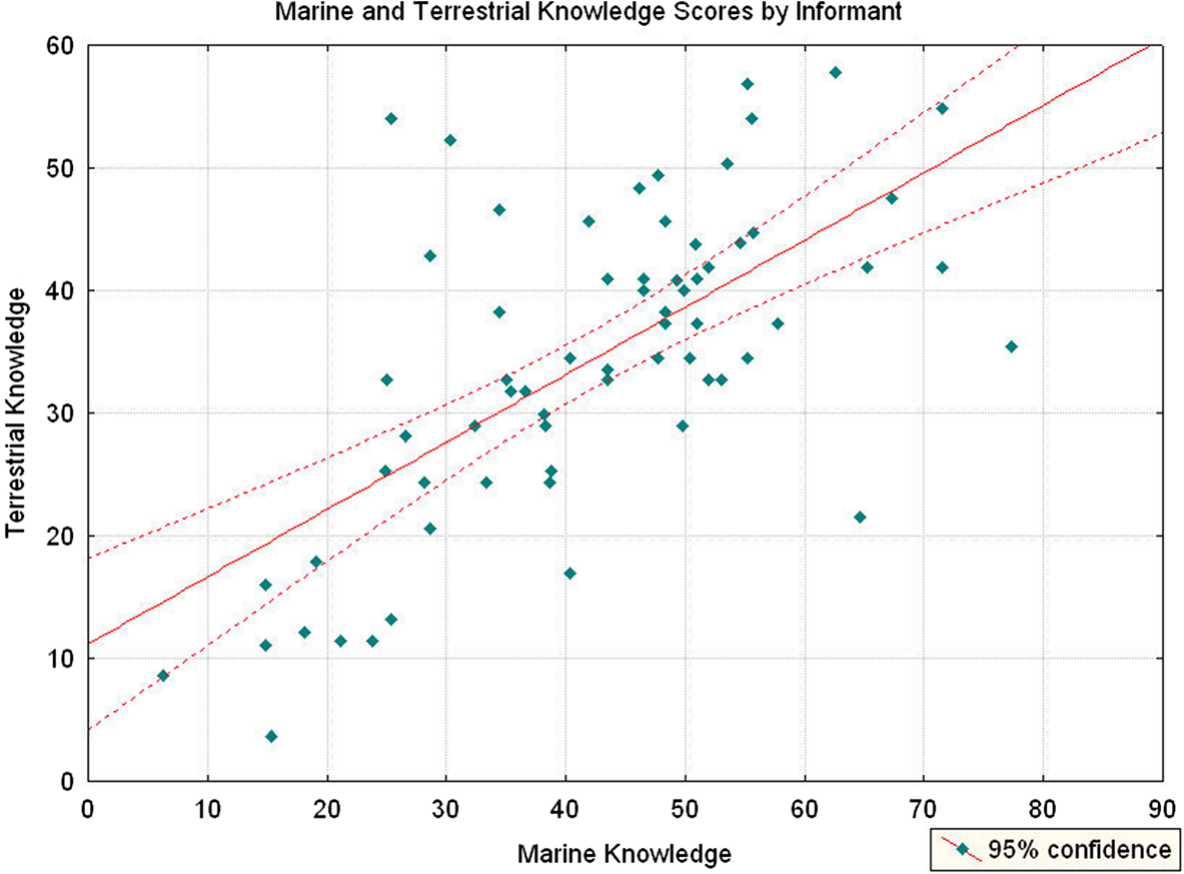
Correlation between terrestrial and marine ethnomedicinal proficiency per informant (r = 0.66)

However, the relationship between these two types of knowledge is not a perfect correlation. This becomes evident by observing (Fig. 4) that 27 % of the participants show ethnomedicinal knowledge scores above fifty percent when tested on marine medicinal knowledge but not when tested on terrestrial medicinal knowledge. Conversely, two collaborators display a certain degree of proficiency in terrestrial medicinal knowledge but their scores languish when faced with marine organisms.

The former suggests that there is a differential acquisition of knowledge. We tested the relationship of three variables; age, years of formal schooling, and gender with ethnomedicinal knowledge proficiency to account for the variation in knowledge acquisition within the Seri society. The relationship of age and years of formal schooling with ethnomedicinal knowledge proficiency was inferred through a correlation analysis (Table 3). Gender induced differences in ethnomedicinal knowledge proficiency were tested under a paired t-test (Table 4).

**Table 3:** Simple correlation matrix for study variables

Variable	Seri ethnobiological knowledge
Age	0.41^a^
Years of formal schooling	−0.49^a^

^a^Marked correlations are significant at p <0.05. N =67 respondents

**Table 4:** Statistical test of significance, t-test, on ethnomedicinal knowledge proficiency by gender

Parameter	Collaborator group	N	Mean	t-value	p-value
Gender	Male	26	33.43482	−2.59212	0.011768^a^
	Female	41	41.37254		

^a^Significant difference (p <0.05); t(0.05) (two tailed)

*N* number of respondents

There is a moderate positive correlation (p < 0.05) between age and ethnomedicinal knowledge proficiency. Years of formal schooling negatively correlate with ethnomedicinal knowledge proficiency.

Women are significantly (p <0.05) more proficient than men when talking about Seri ethnomedicine in general (Table 4). Accordingly, women display better proficiencies when dealing with plant ethnomedicinal knowledge. However, it is rather curious to see that there is no gender difference in the proficiencies of marine ethnomedicinal knowledge when tested separately (Table 5).

**Table 5:** Statistical test of significance, t-test, on differentiated ethnomedicinal knowledge proficiency based on gender differences

Variable	t-test; Gender					
	Mean Female	Mean Male	t-value	p - value	Valid N Female	Valid N Male
Marine Knowledge Proficiency	44.66744	38.42	1.658159	0.102	41	26
Terrestrial knowledge Proficiency	38.07664	28.4485	3.2227	0.00198^a^	41	26

^a^Significant difference (p <0.05); t (0.05) (two tailed)

*N* number of respondents

## Discussion

We argue that it is an erroneous trend in ethnobiology to conceive the ethnomedicinal systems of hunter-gatherers as limited and short. These assumptions are developed under the logic that a foraging mode of subsistence, characterised by low population densities, lack of domesticated livestock, and a nomadic lifestyle will help such societies to flourish in relatively disease-free conditions and as such, they will only develop abbreviated ethnomedicinal systems [123].

Even when it is true that the development of sedentism and the intensification of agriculture [124, 125], along with the shift in diet [126, 127] play an important role in increasing infectious and chronic-degenerative diseases, along with nutritional deficiencies, the daily life of hunter-gatherers is still threatened by parasitic diseases acquired by game and water consumption [128, 129] and gender-associated behaviors [130], snake and insect bites [76, 131], foraging and hunting risks [132], intra- and inter-communal violence [133], and climate challenges [134]. Therefore, we suggest that hunter-gatherer ethnomedicinal systems should be reevaluated in the light of an all-encompassing ethnobiology and new observations shall not be limited to medicinal ethnobotany.

This paper, along with others [135, 136], suggest that the *materia medica* of non-agricultural societies is more complex and diverse than previously suggested. Thus, researchers should not limit themselves to registering the use of indigenous and endemic plant species, but also of various other different items like seaweed, fungi, lichens, insects, marine invertebrates, reptiles, fish and mammals, many of which are used in cleaver ways and shed light into new metabolites and metabolic routes [29, 67].

Regardless of the richness of Seri *materia medica*, it seems that the use of plants is widely preferred over that of algae, halophytes, insects, fungi, or other marine and terrestrial organisms. The aforementioned was first noted when we asked people to free-list all of the Seri medicine they knew. A great majority of the collaborators, whose free-lists averaged 1 min and 16 s (with an exceptional maximum length of 2 min and 3 s), would not mention any marine organism until prompted to do so. Yet, Seri marine medicine contains at least 22 organisms [29], many of which show high cultural salience and bioactive efficacy [9].

It is very likely, as happens with the highly endemic South African Cape flora [135], that the diversity of plant species found in the Sonoran Desert, nearing 2500 species [52], in conjunction with the relative ease of accessibility of terrestrial habitats and easiness of collection invites humans to consider plants as a primary therapy over other types of organisms. The link between the communities and predominancy of easily available remedies within their pharmacopoeias has also been observed by Alves and Rosa [28] who recorded a higher percentage of remedies derived from terrestrial habitats regardless of the preponderant richness of marine and estuarine taxa in the region. Alves and Rosa [100] argue that the greater variety of terrestrial and freshwater habitats increase the participation of terrestrial taxa in medicinal repertoires. While possible, we argue that access and availability to resources plays a more preponderant role in the construction of medicinal repertoires than habitat diversity. Further research based on a landscape ethnoecology approach [137] may add clarity to the participation of diverse taxa in medicinal repertoires.

Seri ethnobiological knowledge reflects the immense biodiversity of the Sonoran Desert, as shown by their extensive and elaborated ethnotaxonomy [47, 74, 76–80, 138, 139] Furthermore, Seri ethnomedicinal knowledge transforms this biodiversity into culturally tangible products as shown by the presence of 3 kingdoms, 6 Phyla, and 15 families represented within the stimuli material. Most prominent uses of the medicinal organisms are against diarrhea, colds, and menstrual problems. Felger and Moser [53, 67], Marroquin [107], and Zolla et al. [101], have already recognized that gastrointestinal diseases have continuously been a major concern for the Seri. The XXI century has seen the seasonal tendencies of illness remain relatively unchanged. In 2008, one of the authors (NEN) had the opportunity to talk to Dr Jesus Antonio Romero Rodríguez, at that time resident medic of the health center at Haxöl Iihom, Sonora. Over his two years of practicing medicine in Desemboque, Dr. Romero has noticed that healthcare demands obey seasonal patterns. Gastrointestinal diseases come with the months of heat (July to August) and the appearance of respiratory diseases is predicted by winter each year. However, Dr. Romero never mentioned feminine ailments to be frequently treated. It is very likely that women take care of these ailments by themselves, which also explains the occurrence of such a high percentage of feminine-exclusive remedies still in force within the Seri pharmacopoeia. Almost a third of the marine ethnomedicines used as visual stimuli is composed by these sorts of remedies.

The use of local remedies to cure respiratory ailments is a common trend in ethnomedicine [27, 100, 139–142]. It is very likely that the remedies used to fight respiratory and gastrointestinal diseases, along with other infectious ailments have pervaded a number of unrelated local pharmacopoeias because their success can be constantly monitored as there is a cause-effect observations are obvious [143]. Greater variation in the distribution of disease categories can be expected for ailments in which the cause-effect relationship between being cured and administering a particular remedy is less obvious.

There are notorious discrepancy between Seri healthcare demands and the most common causes of death, which are hierarchically reported [144] as 1) high blood pressure, 2) tuberculosis, 3) diabetes, 4) kidney disease, 5) aging, 6) cancer, 7) gastrointestinal ailments, and 8) respiratory diseases. These first six causes of dead correspond to those ailments associated with economic and demographic shifts among desert-dwellers [125–127] towards a market economy and a non-traditional diet. However, the adoption of *Rhizophora mangle*, used earlier for treating dysentery [29, 53, 67] *as an anti-diabetes remedy* is an excellent example of Seri ingenuity and desire for innovation. Smith-Monti [99] suggests a series of biochemical mechanisms by which it is very plausible that *R. mangle* may have a real impact as an anti-diabetes medicine.

In total, 12 of the 17 primary uses given to the organisms differ from those previously reported [29, 53, 67]. The vibrant ingenuity within Seri knowledge might as well explain the relatively low numbers for the fidelity level of use for the majority of these organisms. This is especially true for plants, which are accessible within walking distance and their collection is not dependent on traveling to specific areas of the Seriland in dates coinciding with favourable tidal cycles and the availability of fishing gear. In addition, marine ethnomedicines, such as *Atriplex barclayana* (Fl = 93.87) have very specific uses (heals the sting of skates and rays) contingent upon precise situations, i.e., a naive observer will inevitably have to be at the beach in order to be stung by a ray. Thus, the earliest available remedy against this eventuality is conspicuous and easily recognizable plant *A. barclayana*

The moderate correlation (r = 0.41) between ethnomedicinal knowledge proficiency and age may suggests that ethnomedicinal knowledge is still present in the minds of middle aged and senior people, but is not as easily recalled by the younger Seri. These observations are consistent with the recent history of the Seri society, as it is the generation born in the 1960s-1970s that has a) transitioned into relative sedentarism [53], b) fully embraced a market economy [145–147], and c) become less dependent on traditional resources [67]. Yet, those individuals being born in the 1980s and onwards never experienced this transition and have been raised in a livelihood radically different from the one that their parents experienced. Thus, people born before Seri economic integration, around the mid-1950s, are more likely to show better proficiency when faced with their ethnomedicinal resources. On the one hand, and given the historicity of schooling on the Seriland the positive correlation of age and ethnomedicinal knowledge proficiency corresponds to the negative correlation between years of formal schooling and ethnomedicinal knowledge proficiency (r = -0.49). That is, younger generations have greater opportunities to receive formal education. On the other hand, the positive trend of association of age and ethnomedicinal knowledge scores cannot discard the effect of a secular trend [148]. However, other realms of Seri knowledge have also shown a gap between generations and an overall decline in ecological knowledge [149].

Female collaborators were found to be more proficient (p < 0.05) than men in knowing the names and modes of use of Seri ethnomedicines. Females were also more proficient (p <0.05) in naming and correctly describing the use of terrestrial plants. However, there is no significant difference in proficiencies between females and males when faced with marine ethnomedicinal knowledge (p = 0.102). We expected the results to show a differential access to knowledge based on gender roles. Traditionally, Seri men are fishers and hunters and women are gatherers [76, 80, 81, 109, 145]. Nonetheless, the labor divisions by gender are far from being rigid. One of the authors (NEN) personally witnessed the hunting skills of women when chasing rattlesnakes from which they make necklaces out of the vertebrae. Seri women are quite successful and very skilled at hunting, collecting sea pen shells, and processing fish and crustaceans for food [67]. Presently, due to an increasing participation in the formal market, the Seri with most contact with ethnomedicines are women who prepare these as balms, creams, and soaps to sell outside of the Seriland. Women are also the major participants in picking up several fruits, wood, raw materials for their baskets. Furthermore, women take care of the children; therefore, they need to have the ethnomedicinal knowledge at hand. On the contrary, it is men who spend most of the day fishing. Some of their fishing expeditions require them to travel offshore and rely on whatever resources they can find on the midriff islands or the Baja California shore. If it is men who have the marine expertise, why is there no significant difference in marine ethnomedicinal knowledge proficiency when we compare it by gender? First, most of the marine organisms used in Seri ethnomedicine come from the intertidal zone. These organisms are accessible to anyone. During extreme low tide events it is common to see women going into the intertidal zone to collect shells that will later be used as necklace beads and clams for food. The results presented in this paper coincide with previously observed behaviors [150] that it is women who act as primary health care providers and as the most frequent source of healthcare [151, 152]. Thus, it could be argued that Seri women display better overall ethnomedicinal knowledge proficiency because they, practicing as primary health providers, have more opportunities to specialize in the acquisition, preparation and administration of ethnomedicinal resources. In the end, we cannot help but to agree with Krishna-Deb and Haque [153] that every mother is a mini-doctor, particularly in these coastal settings.

## Conclusions

The results found here, in agreement with those obtained by other authors, indicate that coastal and hunter-gatherer ethnomedicinal systems, due to their extensive use of biodiversity are not as limited in scope and detail as previously depicted. More studies in different coastal societies are necessary to increase our understanding of the human adaptation to coastal environments under non-agricultural livelihoods.

In this study we found that gender is strongly associated to the acquisition of ethnomedicinal knowledge. Females know more about Seri ethnomedicine in general, but males are, at least as equally proficient in terms of marine medicine; this is expressed by different patterns of cultural appropriation and reproduction concerning the use of certain species, but is also explained largely by the role that women have as first healthcare providers and most frequent source of healthcare. Age is moderately correlated with ethnomedicinal knowledge acquisition and conversely years of formal schooling may have a negative overall effect in relationship to ethnomedicinal knowledge. Further research on the transmission and acquisition of specific types of local knowledge are needed in order to foster the conservation of Seri biocultural knowledge.

Finally, hunter-gatherers who have historically suffered the pressure of encroachment by food-producing populations [154], currently have to face other strategies of dispossession [155]. The most common means of dispossession in the Mexican Northwest is privatization for mining, recreational hunting, and touristic purposes [156–160]. The results of this study contribute to showing that foraging societies are efficient users of marginal environments. The privatization of these environments will inevitably restrict the access of foraging communities (usually poor and marginalized), to relatively inexpensive and readily available resources that have always been part of their food and health systems. Therefore, there is a need to protect such areas and livelihoods from encroachment by outside interests, while advocating for the right to self-determination of indigenous communities.

## Notes

1. Brent Berlin [31] has described the Seri taxonomical system as anomalous given that the proportion of monotypic to politypic folk genera among the Seri (80:20) corresponds to that of horticulturalists and not hunter-gatherers. The former opens up the possibility to think of the Seri as devolved agriculturalists, whose system of classification lost the subgeneric taxa, reflecting lesser and lesser direct contact with the living world.
2. CONEVAL’s marginalization index considers three socio-economic dimensions: 1) level of education, 2) housing infrastructure, 3) monetary income. and 4) demographic distribution.
